# Changing epidemiology and viral interplay of hepatitis B, C and D among injecting drug user-dominant prisoners in Taiwan

**DOI:** 10.1038/s41598-021-87975-5

**Published:** 2021-04-20

**Authors:** Ming-Ying Lu, Chun-Ting Chen, Yu-Lueng Shih, Pei-Chien Tsai, Meng-Hsuan Hsieh, Chung-Feng Huang, Ming-Lun Yeh, Ching-I Huang, Shu-Chi Wang, Yi-Shan Tsai, Yu-Min Ko, Ching-Chih Lin, Kuan-Yu Chen, Yu-Ju Wei, Po-Yao Hsu, Cheng-Ting Hsu, Tyng-Yuan Jang, Ta-Wei Liu, Po-Cheng Liang, Ming-Yen Hsieh, Zu-Yau Lin, Shinn-Cherng Chen, Jee-Fu Huang, Chia-Yen Dai, Wan-Long Chuang, Ming-Lung Yu, Wen-Yu Chang

**Affiliations:** 1 Hepatobiliary Division, Department of Internal Medicine and Hepatitis Center, Kaohsiung Medical University Hospital, Kaohsiung Medical University, Kaohsiung, Taiwan, ROC; 2 Faculty of Internal Medicine and Hepatitis Research Center, College of Medicine and Center for Cohort Study and Liquid Biopsy, Kaohsiung Medical University, Kaohsiung, Taiwan, ROC; 3 Health Management Center and Department of Community Medicine, Kaohsiung Medical University Hospital, Kaohsiung, Taiwan, ROC; 4 Department of Medical Laboratory Science and Biotechnology, Kaohsiung Medical University, Kaohsiung, Taiwan, ROC; 5 Division of Gastroenterology and Hepatology, Department of Internal Medicine, Tri-Service General Hospital, National Defense Medical Center, Taipei, Taiwan, ROC; 6 Taiwan Liver Research Foundation, Kaohsiung, Taiwan, ROC

**Keywords:** Microbiology, Gastroenterology

## Abstract

The spreading of viral hepatitis among injecting drug users (IDU) is an emerging public health concern. This study explored the prevalence and the risks of hepatitis B virus (HBV), hepatitis C virus (HCV) and hepatitis D virus (HDV) among IDU-dominant prisoners in Taiwan. HBV surface antigen (HBsAg), antibodies to HCV (anti-HCV) and HDV (anti-HDV), viral load and HCV genotypes were measured in 1137(67.0%) of 1697 prisoners. 89.2% of participants were IDUs and none had HIV infection. The prevalence of HBsAg, anti-HCV, dual HBsAg/anti-HCV, HBsAg/anti-HDV, and triple HBsAg/anti-HCV/anti-HDV was 13.6%, 34.8%, 4.9%, 3.4%, and 2.8%, respectively. HBV viremia rate was significantly lower in HBV/HCV-coinfected than HBV mono-infected subjects (66.1% versus 89.9%, adjusted odds ratio/95% confidence intervals [aOR/CI] = 0.27/0.10–0.73). 47.5% anti-HCV-seropositive subjects (n = 396) were non-viremic, including 23.2% subjects were antivirals-induced. The predominant HCV genotypes were genotype 6(40.9%), 1a(24.0%) and 3(11.1%). HBsAg seropositivity was negatively correlated with HCV viremia among the treatment naïve HCV subjects (44.7% versus 72.4%, aOR/CI = 0.27/0.13–0.58). Anti-HCV seropositivity significantly increased the risk of anti-HDV-seropositivity among HBsAg carriers (57.1% versus 7.1%, aOR/CI = 15.73/6.04–40.96). In conclusion, IUDs remain as reservoirs for multiple hepatitis viruses infection among HIV-uninfected prisoners in Taiwan. HCV infection increased the risk of HDV infection but suppressed HBV replication in HBsAg carriers. An effective strategy is mandatory to control the epidemic in this high-risk group.

## Introduction

Blood-borne viral hepatitis B, C, and D are the major causes of hepatocellular carcinoma and end stage liver diseases. It is estimated approximately 15–20% of the adults are carriers of hepatitis B virus (HBV) and 2–5% are infected with hepatitis C virus (HCV) in Taiwan^1, 2^. Anti-HCV positive rate can be as high as 30% in certain endemic areas located in southwestern coast of Taiwan^3^. Hepatitis D virus (HDV) is a defective virus which requires envelope proteins of HBV to assemble a new virion ^4^. The global prevalence of HDV was around 0.8% in general population and 13% in HBV carriers, which contributes rapid progression of end stage liver diseases^5^.

In Taiwan, the prevalence of chronic hepatitis B in the younger generation dropped to less than 1% after implementation the nationwide HBV vaccination program since 1986 ^1, 6^. As an evolution of antiviral therapy from interferon to direct-acting antiviral drugs (DAA), the cure rate of HCV improves from 50% to more than 97% ^7, 8^. The disease burden of HBV and HCV will gradually decrease in general population, but increases in injecting drug users (IDU). The HBV prevalence in IDUs was around 16% in both hospital-based cohort and prisoners ^9, 10^. However, the HCV prevalence rate was up to around 98% in IDUs with immunodeficiency virus (HIV) infection and 89% in IDUs without HIV infection^10^. Similarly, among HBV-seropositive IDUs in Taiwan, the prevalence rate of HDV in subjects with and without HIV infection in hospital-based population was 74.9% and 43.9%, respectively^11^, and 84.2% and 40.0% in prisoners, respectively^12^.

The transmission of hepatitis B, C and D among IDUs has been an emerging public health concern in Taiwan. Approximately 53,000 prisoners are incarcerated out of the 23 million people in Taiwan, and about half of the prisoners serve their sentences because of substance abuse. Nevertheless, only sporadic native studies were conducted on the epidemiological trends of viral hepatitis among this high-risk population yet. The aims of this study were to explore the prevalence of hepatitis B, C and D, to analyze the risk factors of viral hepatitis in HIV-uninfected IDUs-dominant prisoners and to evaluate the status of HCV treatment in the DAA era.

## Methods

### Subjects

A total of 1137 (67.0%) participants among 1697 inmates of Penghu Prison (Agency of Corrections, Ministry of Justice, Taiwan) received the screening for viral hepatitis B, C and D in September 2019. All inmates were routinely examined for human immune-deficiency virus (HIV-1) test when incarcerated in the prison and recheck it annually. HIV-infected prisoners will be transferred to Taichung Prison owing to lack of infection specialist in Penghu Prison. Seropositive for HIV-1 and clinical information regarding antiviral therapy were obtained from the medical records. This study was taken in conformity to the guidelines of the Declaration of Helsinki and approved by the ethics committee of Kaohsiung Medical University Hospital. Written informed consent was obtained from all study participants.

### Biochemical and hepatitis markers analyses

Biochemical analyses were evaluated on a multichannel autoanalyzer (Hitachi Inc, Tokyo, Japan). The algorithm of screening for hepatitis B, C, and D was shown as Supplementary Fig. S1.

### Hepatitis B

The hepatitis B surface antigen (HBsAg), hepatitis B e antigen (HBeAg), antibody to hepatitis e antigen (anti-HBe), antibody to hepatitis B core antigen (anti-HBc IgG) and antibody to hepatitis B surface antigen (anti-HBs) were detected using commercially available enzyme-linked immunosorbent assay (ELISA) kits (Abbott Laboratories, North Chicago, IL, USA). The HBsAg titer was assessed using the Abbott Architect HBsAg QT assay (detection range: 0.05 to 250 IU/ml). HBV DNA was quantified using the CobasAmpliPrep/CobasTaqMan HBV assay (detection range 20–1.7 × 10^8^ IU/ml, CAP/CTM Version 2.0, Roche Diagnostics, Indianapolis, IN) ^13^.

### Hepatitis C

Anti-HCV antibody was determined by the third generation, commercially available ELISA kit (Ax SYM HCV III; Abbott Laboratories, North Chicago, IL). Both the serum HCV RNA and genotypes were assessed by real-time PCR assays (low limit of detection: 12 IU/ml, RealTime HCV; Abbott Molecular, Des Plaines IL, USA)^14^.

### Hepatitis D

Anti‐HDV IgG antibody was determined using the anti‐HDV ELISA kit (General Biologicals Corporation, Taiwan). Serum HDV RNA was quantified using a LightMix Kit HDV (Berlin, Germany) on a Roche LightCycler (detection limit: 10 copies/ml)^15^.

### Statistical analysis

The continuous variables were compared using Student's t test. The categorical variables were accessed using *χ*^2^ or Fisher's exact test. Multivariate logistic regression test was used to analyze the risk factors of virus exposure and viremia. A two-tailed *p*-value < 0.05 was considered statistically significant. Anti-HCV-seropositive subjects with undetectable HCV RNA was defined as spontaneous HCV clearance if they never received anti-HCV therapy, and as treatment-induced HCV clearance if they had ever received anti-HCV therapy. Subjects who were HCV viremic or who were treatment-induced HCV clearance were further classified as supposed HCV viremia. All statistical analyses were performed using Statistical Product and Service Solutions (SPSS) software, version 17.0. (SPSS Inc., Chicago, IL).

## Results

### Basic characteristics

The mean age of 1137 participants was 45.4 ± 9.6 years; 1130 (99.4%) were male; 1014 (89.2%) had an intravenous substance abuse history; None had known history of HIV infection (Table 1).

**Table 1:** Baseline demographics.

	All	HBsAg (+)	Anti-HCV (+)	Anti-HDV (+)	NBNC hepatitis
n	1137	155 (13.6%)	396 (34.8%)	39 (3.4%)	83 (7.3%)
Age (years)	45.4 ± 9.6	46.1 ± 7.4	47.8 ± 8.2	47.7 ± 7.3	41.9 ± 10.4
**Sex**					
Male	1130 (99.4%)	154 (99.4%)	396(100.0%)	39 (100.0%)	82 (98.8%)
Female	7 (0.6%)	1 (0.6%)	0 (0.0%)	0 (0.0%)	1 (1.2%)
AST (IU/ml)	26.3 ± 16.4	28.1 ± 14.2	32.5 ± 23.1	29.1 ± 15.4	38.8 ± 14.9
ALT (IU/ml)	32.3 ± 29.9	35.1 ± 25.8	42.6 ± 41.2	34.8 ± 25.8	63.0 ± 25.3
BMI (kg/m^2^)	24.2 ± 3.5	23.8 ± 3.0	24.0 ± 3.2	24.3 ± 3.1	27.5 ± 4.5
FIB-4	0.95 ± 0.62	1.09 ± 0.81	1.22 ± 0.82	1.32 ± 1.20	0.81 ± 0.64
**IDU**					
Yes	1014 (89.2%)	134 (86.5%)	389 (98.2%)	39 (100.0%)	64 (77.1%)
No	123 (10.8%)	21 (13.5%)	7 (1.8%)	0 (0.0%)	19 (22.9%)
**Co-infection**					
HBsAg (+)	–	–	56(14.1%)	39(100%)	–
Anti-HCV (+)	–	56(36.1%)	–	32(82.1%)	–
Anti-HDV (+)	–	39(25.2%)	32(8.1%)	–	–
All HBV/HCV/HDV markers (+)	–	32(20.6%)	32(8.1%)	32(82.1%)	–
HIV (+)	0 (0.0%)	0 (0.0%)	0 (0.0%)	0 (0.0%)	0 (0.0%)

*HBsAg* hepatitis B surface antigen; *anti-HCV* hepatitis C antibody; *anti-HDV* hepatitis D antibody; *NBNC hepatitis* non-B, non-C hepatitis, defined as subjects with elevated liver function tests but seronegative for HBsAg and anti-HCV; *AST* aspartate aminotransferase; *ALT* alanine aminotransferase; *BMI* body mass index; *FIB-4* Fibrosis-4 index; *IDU* injecting drug user; *HIV* human immunodeficiency virus.

### The prevalence of viral hepatitis

The seroprevalence rate of HBV, HCV, and HDV infections was 13.6%, 34.8% and 3.4%, respectively. Among 155 subjects with HBsAg seropositivity, 56 (36.1%) subjects had anti-HCV seropositivity, 39 (25.2%) with anti-HDV seropositivity, and 32 (20.6%) with triple HBV/HCV/HDV seromarkers. Among 396 subjects with anti-HCV seropositive, 56 (14.1%) had HBV/HCV dual infection, and 32 (8.1%) HBV/HCV/HDV triple infections. The anti-HCV seroprevalence rate increased gradually with age, greater than 35% in groups older than 40 years old (Supplementary Fig. S2). Among 39 subjects with HBV/HDV dual infections, 32 (82.1%) subjects had triple HBV/HCV/HDV infections. The global HBV/HCV/HDV triple infection rate was 2.8%. Eighty-three subjects (7.3%) with elevated liver function test were seronegative for HBsAg and anti-HCV (Table 1).

### Factors associated with HBV infection and HBV viremia

Table 2 showed the factors associated with HBsAg-seropositivity. No HBV carrier was born after 1986, the year of mass HBV vaccination in Taiwan. Among 155 HBV carrier, 9.7% subjects were HBeAg positive, and 89.0% subjects had anti-HBe antibody. The proportions of sex, history of IDU, anti-HCV seropositivity, and the levels of liver function tests were comparable between subjects with and without HBsAg-seropositivity. Compared with HBsAg seronegative subjects, HBsAg seropositive inmates had significantly lower rate of supposed HCV viremia (53.6% vs 79.4%, *p* = 2.9 × 10^–5^) and significantly lower HCV RNA levels (2.29 ± 3.04 vs 3.25 ± 3.05 log_10_IU/ml, *p* = 0.030).

**Table 2:** Factors associated with HBV infection and HBV viremia.

	All (n = 1,137)			HBsAg (+) (n = 155)		
	HBsAg (+)	HBsAg (−)	*p*-value	HBV DNA (+)	HBV DNA (−)	*p*-value
n	155 (13.6%)	982 (86.4%)		126 (81.3%)	29 (18.7%)	
**Age**						
Mean ± SD	46.1 ± 7.4	45.3 ± 9.9	0.227	45.9 ± 7.5	47.0 ± 7.2	0.466
Before 1986	155 (100%)	878 (89.4%)	2.2 × 10^–7^	126 (100%)	29 (100%)	N/A
After 1986	0 (0.0%)	104 (10.6)		0 (0.0%)	0 (0.0%)	
**Sex**						
Male	154 (99.4%)	976 (99.4%)	1.000	125 (99.2%)	29 (100%)	1.000
Female	1 (0.6%)	6 (0.6%)		1 (0.8%)	0 (0.0%)	
AST (IU/ml)	28.1 ± 14.2	26.0 ± 16.7	0.143	27.9 ± 14.4	29.0 ± 13.3	0.691
ALT (IU/ml)	35.1 ± 25.8	31.9 ± 30.5	0.219	35.3 ± 26.2	34.1 ± 24.6	0.821
BMI (kg/m^2^)	23.8 ± 3.0	24.2 ± 3.6	0.173	23.7 ± 2.9	24.3 ± 3.1	0.208
FIB-4	1.09 ± 0.81	0.93 ± 0.58	0.002	1.02 ± 0.59	1.41 ± 1.39	0.156
**IDU**						
Yes	134 (86.5%)	880 (89.6%)	0.239	109 (86.5%)	25 (86.2%)	1.000
No	21 (13.5%)	102 (10.4%)		17 (13.5%)	4 (13.8%)	
NUC (n, %)	6 (3.9%)	N/A	N/A	5 (4.0%)	1 (3.4%)	1.000
**HBeAg in HBsAg(+) subjects**						
Positive	15 (9.7%)	N/A	N/A	13(10.3%)	2 (6.9%)	0.738
Negative	140 (90.3%)			113 (89.7%)	27 (93.1%)	
**Anti-HBe in HBsAg(+) subjects**						
Positive	138 (89.0%)	N/A	N/A	112 (88.9%)	26 (89.7%)	1.000
Negative	17 (11.0%)			14 (11.1%)	3 (10.3%)	
**Anti-HBs**						
Positive	5 (3.2%)	657 (67.2%)	6.4 × 10^–51^	4 (3.2%)	1 (3.4%)	1.000
Negative	150 (96.8%)	321 (32.8%)		122 (96.8%)	28 (96.6%)	
**Anti-HBc**						
Positive	154 (99.4%)	627 (64.2%)	1.1 × 10^–14^	126 (100%)	28 (96.6%)	0.187
Negative	1 (0.6%)	350 (35.8%)		0 (0.0%)	1 (3.4%)	
**Anti-HCV**						
Positive	56 (36.1%)	340 (34.6%)	0.715	37 (29.4%)	19 (65.5%)	2.6 × 10^–4^
Negative	99 (63.9%)	642 (65.4%)		89 (70.6%)	10 (34.5%)	
**HCV RNA in anti-HCV (+) subjects**						
Mean ± SD (log IU/ml)	2.29 ± 3.04	3.25 ± 3.05	0.030	1.88 ± 2.83	3.08 ± 3.34	0.193
Positive	21 (37.5%)	187 (55.0%)	0.015	12 (32.4%)	9 (47.4%)	0.274
Negative	35 (62.5%)	153 (45.0%)		25 (67.6%)	10 (52.6%)	
**Supposed HCV RNA in anti-HCV (+) subjects**						
Positive	30 (53.6%)	270 (79.4%)	2.9 × 10^–5^	17 (45.9%)	13 (68.4%)	0.110
Negative	26 (46.4%)	70 (20.6%)		20 (54.1%)	6 (31.6%)	
**HCV genotype in HCV RNA (+) subjects**						
1a	5(23.8%)	45(23.8%)	0.338	1(8.3%)	4(44.4%)	0.093
1b	2(9.5%)	20(10.6%)		2(16.7%)	0(0.0%)	
2	0(0.0%)	21(11.1%)		0(0.0%)	0(0.0%)	
3	1(4.8%)	22(11.6%)		0(0.0%)	1(11.2%)	
6	13(61.9%)	72(38.1%)		9(75.0%)	4(44.4%)	
Mixed	0(0.0%)	3(1.6%)		0(0.0%)	0(0.0%)	
Undetermined	0(0.0%)	6(3.2%)		0(0.0%)	0(0.0%)	
**Anti-HDV in HBsAg (+) subjects**						
Positive	39(25.2%)	N/A	N/A	26(20.6%)	13(44.8%)	0.007
Negative	116(74.8%)			100(79.4%)	16(55.2%)	
**HDV RNA in anti-HDV (+) subjects**						
Positive	21(53.8%)	N/A	N/A	16(61.5%)	5(38.5%)	0.173
Negative	18(46.2%)			10(38.5%)	8(61.5%)	

p.s. Supposed HCV RNA seropositivity included HCV viremia subjects and HCV non-viremia subjects induced by antiviral therapy.

*AST* aspartate aminotransferase; *ALT* alanine aminotransferase; *BMI* body mass index; *FIB-4* Fibrosis-4 index; *IDU* injecting drug users; *NUC* nucleotide analogue; *HBsAg* hepatitis B surface antigen; *HBeAg* hepatitis B envelope antigen; *anti-HBe* hepatitis B envelop antibody; *anti-HBs* hepatitis B surface antibody; *anti-HBc* hepatitis B core antibody; *HBV DNA* hepatitis B virus deoxyribonucleic acid; *anti-HCV* hepatitis C antibody; *HCV RNA* hepatitis C virus ribonucleic acid ; anti-HDV, hepatitis D antibody; *HDV RNA* hepatitis D virus ribonucleic acid.

Among 155 HBsAg carriers, 126 (81.3%) subjects had detectable HBV DNA. HBV viremic subjects had significantly lower rates of anti-HCV and anti-HDV seropositivity than those with undetectable HBV DNA (anti-HCV positive rate: 29.4% vs 65.5%, *p* = 2.6 × 10^–4^; anti-HDV positive rate: 20.6% vs 44.8%, *p* = 0.007) (Table 2).

Multivariate regression analysis showed HBsAg seropositivity was negatively associated with supposed HCV viremia among all subjects (adjusted OR = 0.29, 95% CI = 0.16–0.53, *p* = 5.5 × 10^–5^). HBV/HCV dual infections had significantly lower rate of HBV viremia among HBsAg-seropositive subjects (adjusted OR = 0.27, 95% CI = 0.10–0.73, *p* = 0.010) (Table 3).

**Table 3:** Multivariate regression analysis of factors associated with hepatitis B infection.

	OR	95% CI	Adjusted *p*-value
**HBsAg seropositivity among all subjects**			
Age (before 1986 vs after 1986)	0.00	0.00-	0.999
FIB-4	1.14	0.82–1.60	0.432
Supposed HCV RNA (positive vs negative)	0.29	0.16–0.53	5.5 × 10^–5^
**HBV viremia among HBsAg (+) subjects**			
Anti-HCV (positive vs negative)	0.27	0.10–0.73	0.010
Anti-HDV (positive vs negative)	0.68	0.25–1.87	0.454

p.s. Supposed HCV RNA seropositivity included HCV viremia subjects and HCV non-viremia subjects induced by antiviral therapy.

*HBsAg* hepatitis B surface antigen; *FIB-4* fibrosis-4 index; *anti-HCV* hepatitis C antibody; *anti-HDV* hepatitis D antibody.

### Factors associated with HCV infection and HCV viremia

**Figure 1:**
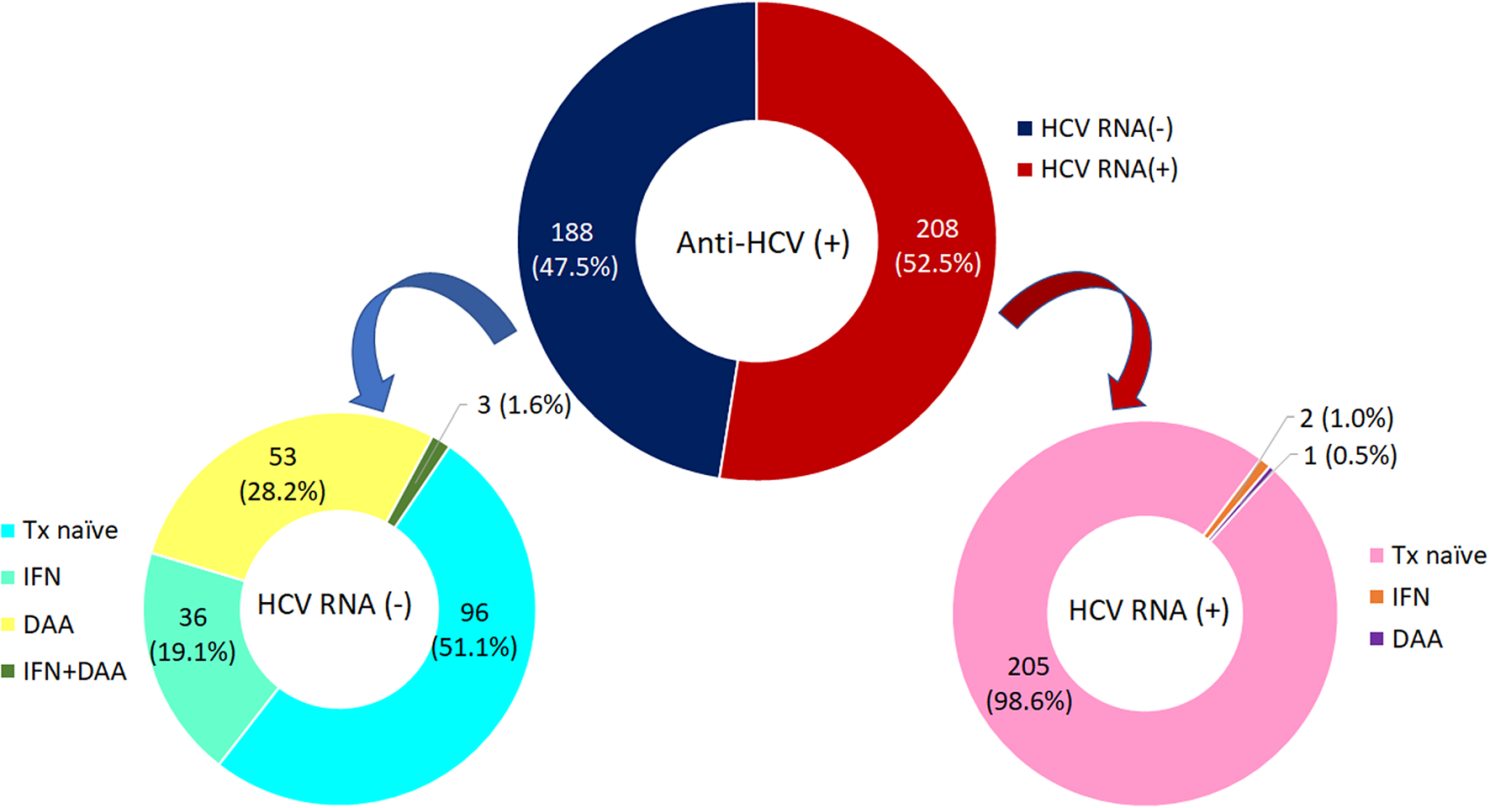
History of treatment among anti-HCV seropositive subjects.

Among 396 anti-HCV positive subjects, 208 subjects had detectable HCV RNA (52.5%), including 3 subjects failed to prior antiviral therapy (interferon n = 2, DAA n = 1). Of 188 HCV non-viremic subjects, 92 subjects (48.9%) were treatment-induced, and 96 subjects (51.1%) were considered spontaneously HCV recovery. Fifty-three subjects (28.2%) were treated with DAA, 36 subjects (19.1%) were treated with interferon, and 3 (1.6%) with DAA after failed to interferon (Fig. 1) The predominant HCV genotype (GT) was GT6 (40.9%), followed by GT1a (24.0%), GT3 (11.1%), GT1b (10.6%), and GT2 (10.1%) (Supplementary Fig. S3).

In univariate analysis, age, levels of AST, ALT, and Fibrosis-4 (FIB-4) index, the proportion of IDU, anti-HBc, and anti-HDV-seropositivity in HBsAg carriers were significantly higher in anti-HCV-seropositive subjects than in anti-HCV-negative subjects. Among HBsAg-seropositive subjects, the proportion of HBV viremia was significantly lower in anti-HCV seropositive subjects than in anti-HCV-seronegative subjects (66.1% vs 89.9%, *p* = 2.6 × 10^–4^). Of 396 anti-HCV-seropositive subjects, the levels of AST, ALT and FIB-4 were significantly higher, but body mass index (BMI) and HBsAg-seropositive rate were significantly lower in the HCV viremia subjects than in those of HCV non-viremic, whatever spontaneous loss of HCV RNA or eradicated by antiviral therapy (Table 4).

**Table 4:** Factors associated with HCV exposure and HCV viremia.

	All (n = 1,137)			Anti-HCV (+) (n = 396)			Treatment naïve Anti-HCV (+)		
	Anti-HCV (+)	Anti-HCV (−)	p-value	HCV RNA (+)	HCV RNA (−)	p-value	HCV RNA (+)	HCV RNA (−) Spontaneous loss	p-value
n	396(34.8%)	741(65.2%)		208(52.5%)	188(47.5%)		205(68.1%)	96(31.9%)	
Age	47.8 ± 8.2	44.1 ± 10.0	6.8 × 10^–11^	48.2 ± 8.4	47.3 ± 8.0	0.261	48.1 ± 8.4	47.4 ± 8.0	0.468
**Sex**									
Male	396(100%)	734(99.1%)	0.103	208(100%)	188(100%)	N/A	205(100%)	96(100%)	N/A
Female	0 (0.0%)	7 (0.9%)		0 (0.0%)	0 (0.0%)		0(0.0%)	0(0.0%)	
AST(IU/ml)	32.5 ± 23.1	23.0 ± 9.7	4.0 × 10^–14^	41.1 ± 27.7	22.9 ± 10.4	1.4 × 10^–16^	41.0 ± 27.8	23.7 ± 13.0	1.7 × 10^–12^
ALT(IU/ml)	42.6 ± 41.2	26.8 ± 19.6	1.8 × 10^–12^	59.4 ± 48.4	24.1 ± 18.2	1.7 × 10^–19^	59.0 ± 48.5	27.0 ± 21.3	3.5 × 10^–14^
BMI (kg/m^2^)	24.0 ± 3.2	24.3 ± 3.6	0.252	23.7 ± 3.2	24.3 ± 3.1	0.046	23.7 ± 3.2	25.0 ± 3.2	0.001
FIB-4	1.22 ± 0.82	0.80 ± 0.41	2.2 × 10^–20^	1.32 ± 0.87	1.12 ± 0.74	0.015	1.32 ± 0.87	1.06 ± 0.59	0.003
**IDU**									
Yes	389(98.2%)	625(84.3%)	6.9 × 10^–13^	203(97.6%)	186(98.9%)	0.453	200(97.6%)	96(100%)	0.182
No	7(1.8%)	116(15.7%)		5(2.4%)	2(1.1%)		5(2.4%)	0(0.0%)	
**HBsAg**									
Positive	56(14.1%)	99(13.4%)	0.715	21(10.1%)	35(18.6%)	0.015	21(10.2%)	26(27.1%)	1.8 × 10^–4^
Negative	340 (85.9%)	642(86.6%)		187(89.9%)	153(81.4%)		184(89.8%)	70(72.9%)	
**HBeAg in HBsAg (+) subjects**									
Positive	5(8.9%)	10(10.1%)	0.813	2(9.5%)	3(8.6%)	1.000	2(9.5%)	3(11.5%)	1.000
Negative	51(91.1%)	89(89.9%)		19(90.5%)	32(91.4%)		19(90.5%)	23(88.5%)	
**Anti-HBe in HBsAg (+) subjects**									
Positive	47(83.9%)	91(91.9%)	0.126	18(85.7%)	29(82.9%)	1.000	18(85.7%)	21(80.8%)	0.715
Negative	9(16.1%)	8 (8.1%)		3(14.3%)	6(17.1%)		3(14.3%)	5(19.2%)	
**Anti-HBs**									
Positive	238(60.1%)	424(57.5%)	0.403	129(62.0%)	109(58.0%)	0.412	127(62.0%)	52(54.2%)	0.200
Negative	158(39.9%)	313(42.5%)		79(38.0%)	79(42.0%)		78((38.0%)	44(45.8%)	
**Anti-HBc**									
Positive	324(82.0%)	457(62.0%)	3.9 × 10^–12^	175(84.1%)	149(79.7%)	0.250	172(83.9%)	79(83.2%)	0.871
Negative	71(18.0%)	280(38.0%)		33(15.9%)	38(20.3%)		33(16.1%)	16(16.8%)	
**HBV DNA in HBsAg (+) subjects**									
Mean ± SD (log IU/ml)	2.30 ± 2.07	3.29 ± 1.87	0.003	1.82 ± 2.10	2.59 ± 2.03	0.178	1.82 ± 2.10	2.85 ± 2.02	0.093
Positive	37(66.1%)	89(89.9%)	2.6 × 10^–4^	12(57.1%)	25(71.4%)	0.274	12(57.1%)	20(76.9%)	0.148
Negative	19(33.9%)	10(10.1%)		9(42.9%)	10(28.6%)		9(42.9%)	6(23.1%)	
**Anti-HDV in HBsAg (+) subjects**									
Positive	32(57.1%)	7(7.1%)	5.1 × 10^–12^	10(47.6%)	22(62.9%)	0.265	10(47.6%)	17(65.4%)	0.221
Negative	24(42.9%)	92(92.9%)		11(52.4%)	13(37.1%)		11(52.4%)	9(34.6%)	
**HDV RNA in anti-HDV **(+)** subjects**									
Positive	18(56.3%)	3(42.9%)	0.682	4(40.0%)	14(63.6%)	0.267	4(40.0%)	12(70.6%)	0.224
Negative	14(43.8%)	4(57.1%)		6(60.0%)	8(36.4%)		6(60.0%)	5(29.4%)	

*HCV* hepatitis C virus; *Anti-HCV* hepatitis C antibody; *HCV RNA* hepatitis C ribonucleic acid ; *AST* aspartate aminotransferase; *ALT* alanine aminotransferase; *BMI* body mass index; *FIB-4* Fibrosis-4 index; *IDU* injecting drug user; *HBsAg* hepatitis B surface antigen; *HBeAg* hepatitis B envelope antigen; *anti-HBe* hepatitis B envelop antibody; *anti-HBs* hepatitis B surface antibody; *anti-HBc* hepatitis B core antibody; *HBV DNA* hepatitis B virus deoxyribonucleic acid; anti-HDV, hepatitis D antibody; *HDV RNA* hepatitis D virus ribonucleic acid.

p.s. Supposed HCV RNA seropositivity included HCV viremia subjects and HCV non-viremia subjects induced by antiviral therapy.

In multivariate regression analysis, IDU (adjusted OR = 18.45, 95% CI = 1.41–241.76, p = 0.026) and anti-HDV seropositivity (adjusted OR = 12.25, 95% CI = 4.85–30.93, *p* = 1.1 × 10^–7^) was positively associated with anti-HCV seropositivity. HBV viremia was negatively associated with anti-HCV seropositivity (adjusted OR = 0.31, 95% CI = 0.11–0.86, p = 0.024). BMI and HBsAg seropositivity were significantly negatively correlated with HCV viremia, whatever among all anti-HCV-seropositive subjects or among treatment-naïve anti-HCV-seropositive subjects (Table 5).

**Table 5:** Multivariate regression analysis of factors associated with HCV infection.

	OR	95% CI	Adjusted *p*-value
**Anti-HCV seropositivity among all subjects**			
Age	0.98	0.92–1.04	0.543
AST	1.03	0.94–1.12	0.520
ALT	1.00	0.96–1.05	0.996
FIB-4	1.02	0.45–2.33	0.955
IDU (yes vs no)	18.45	1.41–241.76	0.026
Anti-HBc (positive vs negative)	0.00	0.00-	1.000
HBV DNA (positive vs negative)	0.31	0.11–0.86	0.024
Anti-HDV (positive vs negative)	12.25	4.85–30.93	1.1 × 10^–7^
**HCV viremia among anti-HCV (+) subjects**			
AST	1.03	0.97–1.09	0.363
ALT	1.05	1.02–1.08	0.002
BMI	0.89	0.83–0.97	0.004
FIB-4	0.66	0.43–1.01	0.056
HBsAg (positive vs negative)	0.42	0.21–0.85	0.016
**HCV viremia among treatment naïve anti-HCV (+) subjects**			
AST	1.02	0.95–1.10	0.605
ALT	1.04	1.00–1.08	0.060
BMI	0.86	0.79–0.94	0.001
FIB-4	0.84	0.44–1.60	0.589
HBsAg (positive vs negative)	0.27	0.13–0.58	6.9 × 10^–4^

*HCV* hepatitis C virus; *anti-HCV* hepatitis C antibody; *AST* aspartate aminotransferase; *ALT* alanine aminotransferase; *BMI* body mass index; *FIB-4* Fibrosis-4 index; *IDU* injecting drug users; *HBsAg* hepatitis B surface antigen; *anti-HBc* hepatitis B core antibody; *HBV DNA* hepatitis B virus deoxyribonucleic acid; *anti-HDV* hepatitis D antibody.

### Factors associated with HDV infection and HDV viremia

Among the HBsAg carriers, none of anti-HDV-seropositive subjects was born after 1986. In univariate analysis, IDU (100% vs 81.9%, *p* = 0.002) and the anti-HCV positive rate (82.1% vs 20.7%, *p* = 5.2 × 10^–12^) were significantly higher, whereas the HBV DNA level was significantly lower (2.10 ± 1.79 vs 3.22 ± 1.99 log_10_IU/ml, *p* = 0.002) in HBV/HDV dual infected subjects than in HBV mono-infected subjects. Multivariate regression analysis showed that anti-HCV seropositivity was significantly higher risk of anti-HDV-seropositivity among HBsAg carriers (adjusted OR = 15.73, 95% CI = 6.04–40.96, *p* = 1.7 × 10^–8^).

Among 39 HBV/HDV dual infected subjects, 21 (53.8%) had detectable HDV RNA. Among 26 HBV/HDV dual infected subjects with HBV viremia, 16 (61.5%) had detectable HDV RNA. None of the factors listed in Table 6 was associated with HDV viremia in both groups.

**Table 6:** Factors associated with HDV exposure and HDV viremia among HBV carriers.

	HBsAg (+)			HBsAg (+) & Anti-HDV (+)			HBV DNA (+) & anti-HDV (+)		
	Anti-HDV (+)	Anti-HDV (−)	p-value	HDV RNA (+)	HDV RNA (−)	p-value	HDV RNA (+)	HDV RNA (−)	p-value
n	39(25.2%)	116(74.8%)		21(53.8%)	18(46.2%)		16(61.5%)	10(38.5%)	
**Age**									
Mean ± SD	47.7 ± 7.3	45.5 ± 7.4	0.108	47.5 ± 7.5	48.0 ± 7.3	0.843	47.7 ± 7.8	47.2 ± 7.0	0.873
Before 1986	39(100%)	116(100%)	N/A	21(100%)	18(100%)	N/A	16(100%)	10(100%)	N/A
After 1986	0(0.0%)	0(0.0%)		0(0.0%)	0(0.0%)		0(0.0%)	0(0.0%)	
**Sex**									
Male	39(100%)	115(99.1%)	1.000	21(100%)	18(100%)	N/A	16(100%)	10(100%)	N/A
Female	0(0.0%)	1(0.9%)		0(0.0%)	0(0.0%)		0(0.0%)	0(0.0%)	
AST (IU/ml)	29.1 ± 15.4	27.7 ± 13.8	0.606	29.0 ± 13.9	29.3 ± 17.3	0.948	26.6 ± 11.8	29.2 ± 22.3	0.703
ALT (IU/ml)	34.8 ± 25.8	35.1 ± 25.9	0.949	35.3 ± 25.6	34.3 ± 26.8	0.905	32.1 ± 22.3	34.2 ± 31.3	0.840
BMI (kg/m^2^)	24.3 ± 3.1	23.7 ± 2.9	0.276	24.0 ± 3.0	24.5 ± 3.3	0.639	23.5 ± 2.6	24.6 ± 3.0	0.361
FIB-4	1.32 ± 1.20	1.02 ± 0.62	0.140	1.43 ± 1.54	1.20 ± 0.64	0.566	1.09 ± 0.51	1.06 ± 0.63	0.892
**IDU**									
Yes	39(100%)	95(81.9%)	0.002	21(100%)	18(100%)	N/A	16(100%)	10(100%)	N/A
No	0(0.0%)	21(18.1%)		0(0.0%)	0(0.0%)		0(0.0%)	0(0.0%)	
NUC (n, %)	1(2.6%)	5(4.3%)	1.000	0 (0.0%)	1 (5.6%)	0.462	0 (0.0%)	1(10.0%)	0.385
**HBeAg**									
Positive	2(5.1%)	13(11.2%)	0.359	2(9.5%)	0(0.0%)	0.490	1(6.3%)	0(0.0%)	1.000
Negative	37(94.9%)	103(88.8%)		19(90.5%)	18(100%)		15(93.7%)	10(100%)	
**Anti-HBe**									
Positive	35(89.7%)	103(88.8%)	1.000	17(81.0%)	18(100%)	0.110	13(81.3%)	10(100%)	0.262
Negative	4(10.3%)	13(11.2%)		4(19.0%)	0(0.0%)		3(18.7%)	0(0.0%)	
**Anti-HBs**									
Positive	0(0.0%)	5(4.3%)	0.331	0(0.0%)	0(0.0%)	N/A	0(0.0%)	0(0.0%)	N/A
Negative	39(100%)	111(95.7%)		21(100%)	18(100%)		16(100%)	10(100%)	
**Anti-HBc**									
Positive	39(100%)	115(99.1%)	1.000	21(100%)	18(100%)	N/A	16(100%)	10(100%)	N/A
Negative	0(0.0%)	1(0.9%)		0(0.0%)	0(0.0%)		0(0.0%)	0(0.0%)	
**HBV DNA**									
Mean ± SD (log IU/ml)	2.10 ± 1.79	3.22 ± 1.99	0.002	2.25 ± 1.65	1.92 ± 1.98	0.578	2.95 ± 1.19	3.46 ± 1.23	0.305
Positive	26(66.7%)	100(86.2%)	0.007	16(76.2%)	10(55.6%)	0.173	16(100%)	10(100%)	N/A
Negative	13(33.3%)	16(13.8%)		5(23.8%)	8(44.4%)		0(0.0%)	(0.0%)	
**Anti-HCV**									
Positive	32(82.1%)	24(20.7%)	5.2 × 10^–12^	18(85.7%)	14(77.8%)	0.682	13(81.3%)	7(70.0%)	0.644
Negative	7(17.9%)	92(79.3%)		3(14.3%)	4(22.2%)		3(18.7%)	3(30.0%)	
**HCV RNA in anti-HCV (+) subjects**									
Mean ± SD (log IU/ml)	1.82 ± 2.81	2.91 ± 3.28	0.197	1.20 ± 2.37	2.62 ± 3.20	0.180	1.67 ± 2.66	1.58 ± 2.81	0.949
Positive	10(31.3%)	11(45.8%)	0.265	4(22.2%)	6(42.9%)	0.267	4(30.8%)	2(28.6%)	1.000
Negative	22(68.8%)	13(54.2%)		14(77.8%)	8(57.1%)		9(69.2%)	5(71.4%)	
**Supposed HCV RNA in anti-HCV (+) subjects**									
Positive	15(46.9%)	15(62.5%)	0.246	6(33.3%)	9(64.3%)	0.082	5(38.5%)	4(57.1%)	0.642
Negative	17(53.1%)	9(37.5%)		12(66.7%)	5(35.7%)		8(61.5%)	3(42.9%)	
**HCV genotype in HCV RNA (+) subjects**									
1a	2(20.0%)	3(27.3%)	0.745	0(0.0%)	2(33.3%)	0.290	0(0.0%)	0(0.0%)	1.000
1b	1(10.0%)	1(9.1%)		1(25.0%)	0(0.0%)		1(25.0%)	0(0.0%)	
2	0(0.0%)	0(0.0%)		0(0.0%)	0(0.0%)		0(0.0%)	0(0.0%)	
3	1(10.0%)	0(0.0%)		0(0.0%)	1(16.7%)		0(0.0%)	0(0.0%)	
6	6(60.0%)	7(63.6%)		3(75.0%)	3(50.0%)		3(75.0%)	2(100%)	

p.s. Supposed HCV RNA seropositivity included HCV viremia subjects and HCV non-viremia subjects induced by antiviral therapy.

*HDV* hepatitis D virus; *anti-HDV* hepatitis D antibody; *HDV RNA* hepatitis D virus ribonucleic acid; *AST* aspartate aminotransferase; *ALT* alanine aminotransferase; *BMI* body mass index; *FIB-4* Fibrosis-4 index; *IDU* injecting drug user; *NUC* nucleotide analogue; *HBsAg* hepatitis B surface antigen; *HBeAg* hepatitis B envelope antigen; *anti-HBe* hepatitis B envelop antibody; *anti-HBs* hepatitis B surface antibody; *anti-HBc* hepatitis B core antibody; *HBV DNA* hepatitis B virus deoxyribonucleic acid; *anti-HCV* hepatitis C antibody; *HCV RNA* hepatitis C virus ribonucleic acid.

## Discussion

This study revealed the prevalence of hepatitis B, C, and D in Penghu Prison was 13.6%, 34.8%, 3.4%, respectively. IDU was significantly associated with HCV and HDV, but not HBV infection. The prevalence rates of HBV/HCV, HBV/HDV dual infections and HBV/HCV/HDV triple infections was 5%, 3.4% and 2.8%, respectively. The predominant HCV genotypes in IDUs were GT6 (40.9%), GT1a (24.0%) and GT3 (11.1%). The risk of HBV viremia was significantly reduced in the anti-HCV seropositive subjects. HBsAg seropositive and body mass index were negatively correlated with HCV viremia among the treatment naïve HCV subjects. Positive for anti-HCV antibody significantly increased the risk of HDV infection.

In Taiwan, the implementation of universal hepatitis B vaccination since 1986 has substantially decreased the HBsAg carrier rate from–15% to < 1% in young adult ^6^. This study showed the prevalence of hepatitis B among IDUs was similar to that of the general population. An outbreak of HIV infection among IDUs occurred in Taiwan between 2004 and 2006. In this outbreak, the HCV infection rate was up to 98% in HIV-infected IDUs, and some unprecedented HCV genotypes (e.g., 6 g, 6 k, 6w…) had been identified in Taiwan^16^. The prevalence of anti-HCV seropositive was not only extremely high in HIV-infected IDUs (98.7%) but also in HIV-uninfected IDUs (83%) ^10^. Elevated HDV seroprevalence among HBV carriers was simultaneously observed in both HIV-infected IDUs (74.9–84.2%) and HIV-uninfected IDUs (40.0–66.7%) ^11, 12, 17^ To avoid the spread of this epidemic, the Taiwan Centers for Disease Control (CDC) has implemented harm reduction project for IDUs since 2005. The core interventions include the disposable syringe practices, methadone maintenance treatment, HIV counseling, antiretroviral therapy, and public health education programs. Our study revealed the prevalence of HCV and HBV/HDV dual infection among the inmates in Penghu Prison had steadily declined to 34.8% and 25.2% in 2019 (Supplementary Fig. S4 and Table S1).

The superinfection of viral hepatitis among IDUs is common because of the same routes of transmission. Subjects with dual infection have a greater risk of advanced liver disease, cirrhosis and HCC compared with mono-infected subjects ^18, 19^. Our survey revealed the presence of HCV RNA significantly reduced the risk of HBsAg seropositive. Positive for anti-HCV antibody significantly decreased the risk of HBV viremia. In addition, HBV/HDV dual infected subjects had a less HBV DNA level compared with HBV mono-infected subjects. The previous studies revealed HCV exhibited stronger inhibitory action among the subjects with HBV/HCV dual infection^20–22^. HBV reactivation occurs frequently in HBV/HCV coinfected patients receiving DAA therapy but is rare among patients with resolved HBV infection^20, 21^. HDV appeared to be the predominant virus in either HBV/HDV dual infection or in HBV/HCV/HDV triple infection^23, 24^. The complex interplay among these viruses remains poorly understood. Some possible mechanism had been elucidated. (1) Direct inhibition HBV replication by HCV core protein; (2) Eradication of one virus provides available replication space for another; (3) Loss of host immune responses to one virus may improve replication of the other ^25^. Murai et al. found HCV infection suppresses HBV replication via the RIG-1 (retinoic acid-inducible gene-1) like helicase pathway^26^. MicroRNAs play a role in facilitation of HCV dominance as well. miR-122 can restrain HBV replication through cyclin G1-mediated P53 activity ^27^. In contrast, miR-122 stabilizes HCV genome by protecting the 5′ terminus of the HCV RNA from degradation by the host exonuclease ^28^. In addition, HDV can suppress HBV replication via inhibition the host DNA-dependent RNA polymerase involving HBV transcription ^29^. Small hepatitis delta antigen exerts a strong inhibition of HBV mRNAs synthesis or stability^30, 31^. HDV proteins inhibit HBV replication by repressing HBV enhancers and by activating the IFN-α-inducible MxA (myxovirus resistance A) gene^32^.

The distribution of HCV genotypes varies by risk groups and geographically. This study showed the predominant HCV genotypes among the inmates of Penghu Prison were GT6 (40.9%), followed by GT1a (24.0%), and GT3 (11.1%). The previous studies reported the proportion of HCV genotype 6 (28.0–43.4%), 1a (14.9–29.2%), and 3a (7.8–20.2%) was apparently higher among IDUs compared with the general population (GT6: 0–0.49%; GT1a: 0–2.7%; GT3a: 0–0.98%) in Taiwan^3, 10, 16, 33, 34^. Phylogenetic analysis revealed that these distinct subtypes were derived from the spread of HCV along the drug trafficking routes via Vietnam and Thailand^35, 36^. Genotype 1a was common among IDUs in Europe and America as well ^37, 38^. In general, the distribution of HCV genotypes among IDUs in Taiwan did not alter tremendously in the past fifteen years.

**Figure 2:**
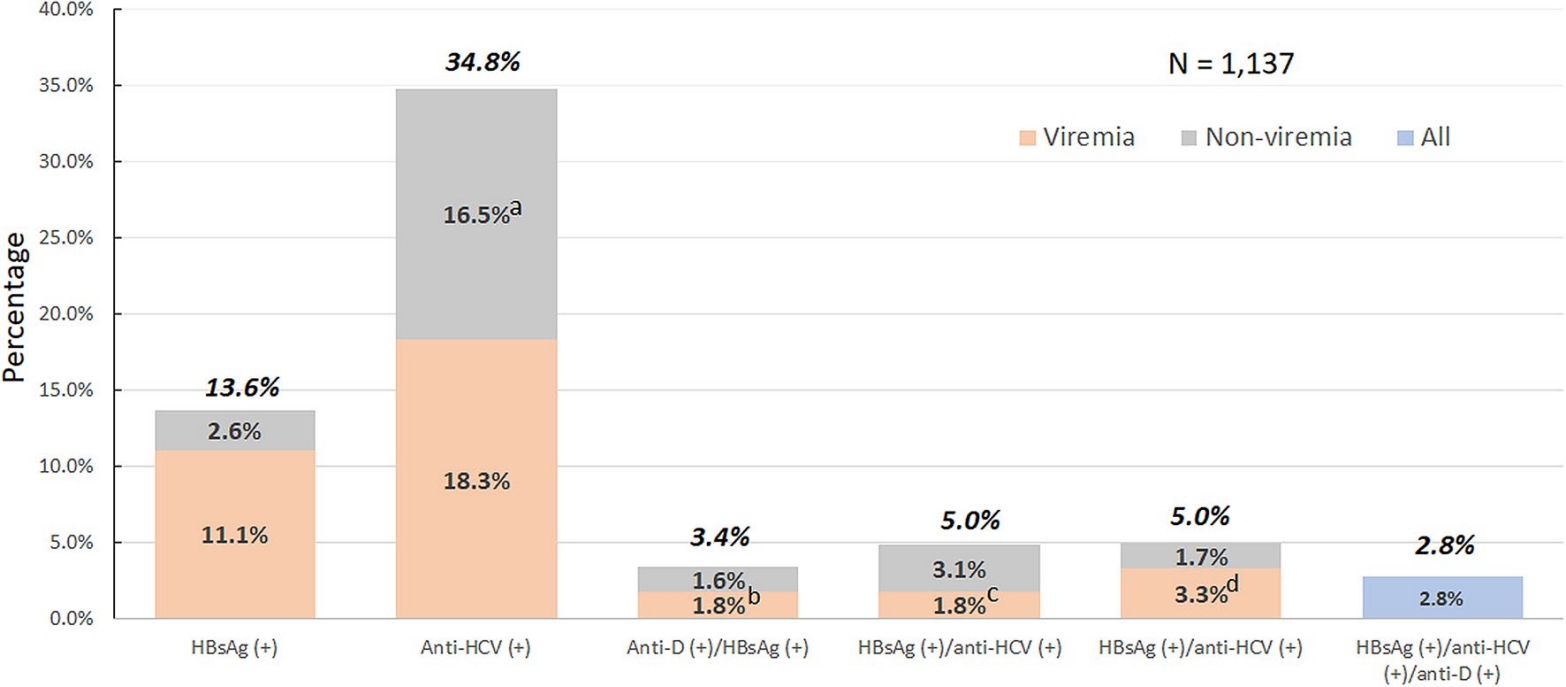
The prevalence of HBV, HCV and HDV infection among prisoners. *HBsAg* hepatitis B virus surface antigen; *anti-HCV* antibodies to hepatitis C virus; *anti-D* antibodies to hepatitis D virus. Viremia indicates seropositive for each corresponding viral DNA or RNA. (**a**) 8.1% were treatment-induced non-viremic; (**b**) HDV viremia; (**c**) HCV viremia; d, HBV viremia.

Dual and/or triple infections of HBV, HCV and/or HDV have been associated with poor long-term outcome^39, 40^. The estimated prevalence rate of anti-HDV was 4.5% among HBsAg-positive carriers and around 0.16% in general population with a total of 12 million people seropositive for anti-HDV worldwide^39^. However, there is no global estimate for HBV/HCV/HDV triple infections available. HCV infection has been associated with risk of HDV infection among HBV carriers in previous study as well as in the current study^11, 39^. Similar to previous study in general population and high-risk groups (PWID and/or HIV-positive patients) in Taiwan^11^, around 80% of HBV/HDV-infected subjects were seropositive for anti-HCV in the current study, with a 2.8% prevalence rate of HBV/HCV/HDV triple infections in the prisoners (Fig. 2).

In this study, 31.7% (95/300) chronic hepatitis C patient concomitant with HCV RNA seropositive had ever received antiviral therapy. The sustained virologic response rate was 87.8% (36/41) in interferon group and 98.2% (56/57) in DAA group, respectively. Asians have a higher likelihood of achieving an interferon-induced viral clearance than their Caucasians counterparts due to carriage of favorable interleukin-28 genotype^41^. With the recommended “standard interferon dose and duration regimens”, SVR is achieved in Asia for around 70% of HCV genotype 1 (GT1) infected cases, approximately 90% of GT2/3, 65% of GT4, and 80% of GT6 patients^42^. The treatment efficacy in HCV infected prisoners was comparable with that in the community cohort.

Although treatment efficacy of the DAA is excellent, there is still a wide gap between the awareness of hepatitis C and access to antiviral therapy among this high-risk population. Irrespective of coinfection with HIV or not, the carrier rate of viral hepatitis is still high among IDUs. In addition to HIV, we recommend the viral hepatitis markers should be examined routinely for all the incarcerated IDUs in Taiwan prisons. The incarcerated inmates may face numerous barriers to health care services. It is necessary to adopt integrated strategies to prevent intravenous drug use, and to assist subjects searching for medical care aggressively. Apart from medical service, social-economic support is important to prevent the incarcerated IDUs from reuse intravenous drugs after being released.

There are three major pillars for controlling the epidemic of viral hepatitis in this high-risk groups. Firstly, universal vaccination with HBV vaccine, which could provide protection from HBV and HDV infection for HBV-unexposed subjects^43^; secondly, education for avoiding percutaneous risk behaviors, such as abstinence of drugs, harm reduction with safe needles, and safe sex; thirdly, treatment as prevention. It is critical in particular for HCV control since there are no vaccine available. The concept of HCV micro-elimination could help prevent the spreading of HCV in the high-risk groups^44^. We recently approved the concept that an outreach screening program with onsite DAA treatment is the key toward HCV micro-elimination in uremic HCV patients under maintenance hemodialysis^45, 46^. Therefore, an outreach onsite HCV treatment program is proposed for HCV micro-elimination in the high risk setting.

There are limitations in our study. This was a cross-sectional study. It failed to find out the change of molecular epidemiology of hepatitis B, C and D over time. However, it is very difficult to follow the inmates when they are released or transferred. Since there is no HIV-infected subject in Penghu Prison, the results may not fully reflect the actual status of viral hepatitis among IDUs in Taiwan. Because of the highly coexistence between the viral hepatitis and HIV, the infection rate of viral hepatitis among IDUs may be underestimated.

## Conclusion

This study revealed a marked decline in the prevalence of HBV, HCV, and HDV among HIV-uninfected IDUs over the past fifteen years in Taiwan. Although the universal HBV vaccination program and disposable syringe practices indeed prevent the spread of viral hepatitis, IDUs still emerge as a reservoir for viral hepatitis in Taiwan. Despite high efficacy DAAs been on the market for several years, there is a wide gap between awareness of viral hepatitis and antiviral therapy among IDUs. More effective public health policy is required to eliminate the epidemic in these high-risk groups.

## Supplementary Information

Supplementary Information.

## Data Availability

The datasets generated during and/or analysed during the current study are available from the corresponding author on reasonable request.
