# Supplementary material for: Changing epidemiology and viral interplay of hepatitis B, C and D among injecting drug user-dominant prisoners in Taiwan
Source: Sci Rep. 2021 Apr 20;11:8554. doi: 10.1038/s41598-021-87975-5 (PMC8058093; doi:10.1038/s41598-021-87975-5)
Supplement: Supplementary file 1 — Supplementary Information. [file 41598_2021_87975_MOESM1_ESM.docx]

**Changing epidemiology and viral interplay of hepatitis B, C and D among injecting drug user-dominant prisoners in Taiwan**

Ming-Ying Lu^1^, Chun-Ting Chen^5^, Yu-Lueng Shih^5^, Pei-Chien Tsai^1^, Meng-Hsuan Hsieh^1,3^, Chung-Feng Huang^1,2^, Ming-Lun Yeh^1,2^, Ching-I Huang^1,2^, Shu-Chi Wang^4^, Yi-Shan Tsai^1^, Yu-Min Ko^1^, Ching-Chih Lin^1^, Kuan-Yu Chen^1^, Yu-Ju Wei^1^ , Po-Yao Hsu^1^; Cheng-Ting Hsu^1^; Tyng-Yuan Jang^1^, Ta-Wei Liu^1^, Po-Cheng Liang^1^, Ming-Yen Hsieh^1^, Zu-Yau Lin^1,2^, Shinn-Cherng Chen^1,2^, Jee-Fu Huang^1,2^, Chia-Yen Dai^1,2,3^, Wan-Long Chuang^1,2^, Ming-Lung Yu^1,2*^, Wen-Yu Chang^1,6^

**Affiliations**

^1^Hepatobiliary Division, Department of Internal Medicine, Kaohsiung Medical University Hospital, Kaohsiung Medical University, Kaohsiung, Taiwan
^2^Faculty of Internal Medicine and Hepatitis Research Center, College of Medicine and Center for Cancer Research and Center for Cohort Study and Liquid Biopsy, Kaohsiung Medical University, Kaohsiung, Taiwan

^3^Health Management Center and Department of Community Medicine, Kaohsiung Medical University Hospital, Kaohsiung, Taiwan

^4^Department of Medical Laboratory Science and Biotechnology, Kaohsiung Medical University, Kaohsiung, Taiwan

^5^Division of Gastroenterology and Hepatology, Department of Internal Medicine, Tri-Service General Hospital, National Defense Medical Center, Taipei, Taiwan

^6^Taiwan Liver Research Foundation, Taiwan

***Corresponding author: Ming-Lung Yu, M.D, Ph.D.**

Address: Hepatobiliary Division, Department of Internal Medicine, Kaohsiung Medical University Hospital, No.100, Shin-Chuan 1st Road, Sanmin Dist., Kaohsiung City, 80708, Taiwan

Tel: +886-7-312-1101 ext. 7475

Fax: +886-7-312-3955

E-mail: [fish6069@gmail.com](mailto:fish6069@gmail.com)

Supplementary figure 1. Algorithm of screening for hepatitis B, C and D


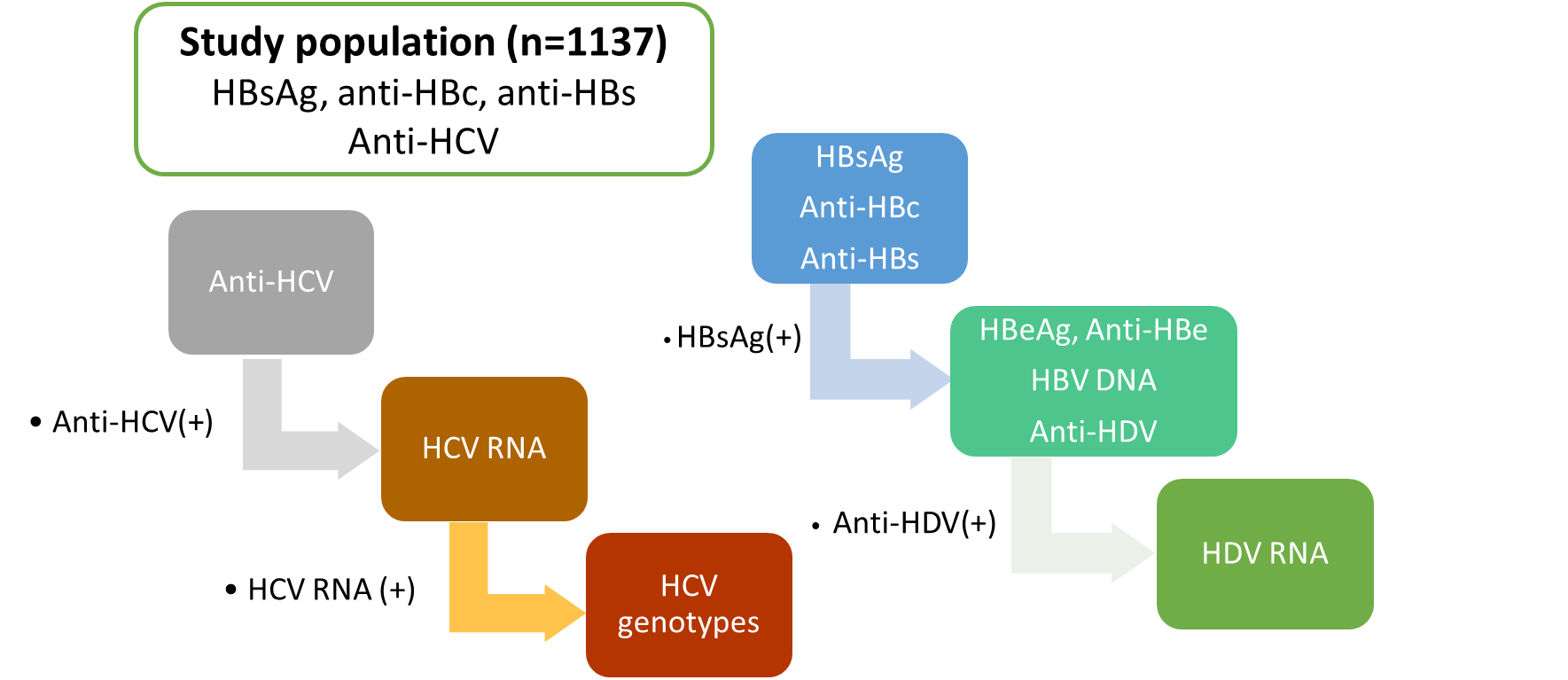


Supplementary figure 2. The anti-HCV seropositive rate in Penghu Prison


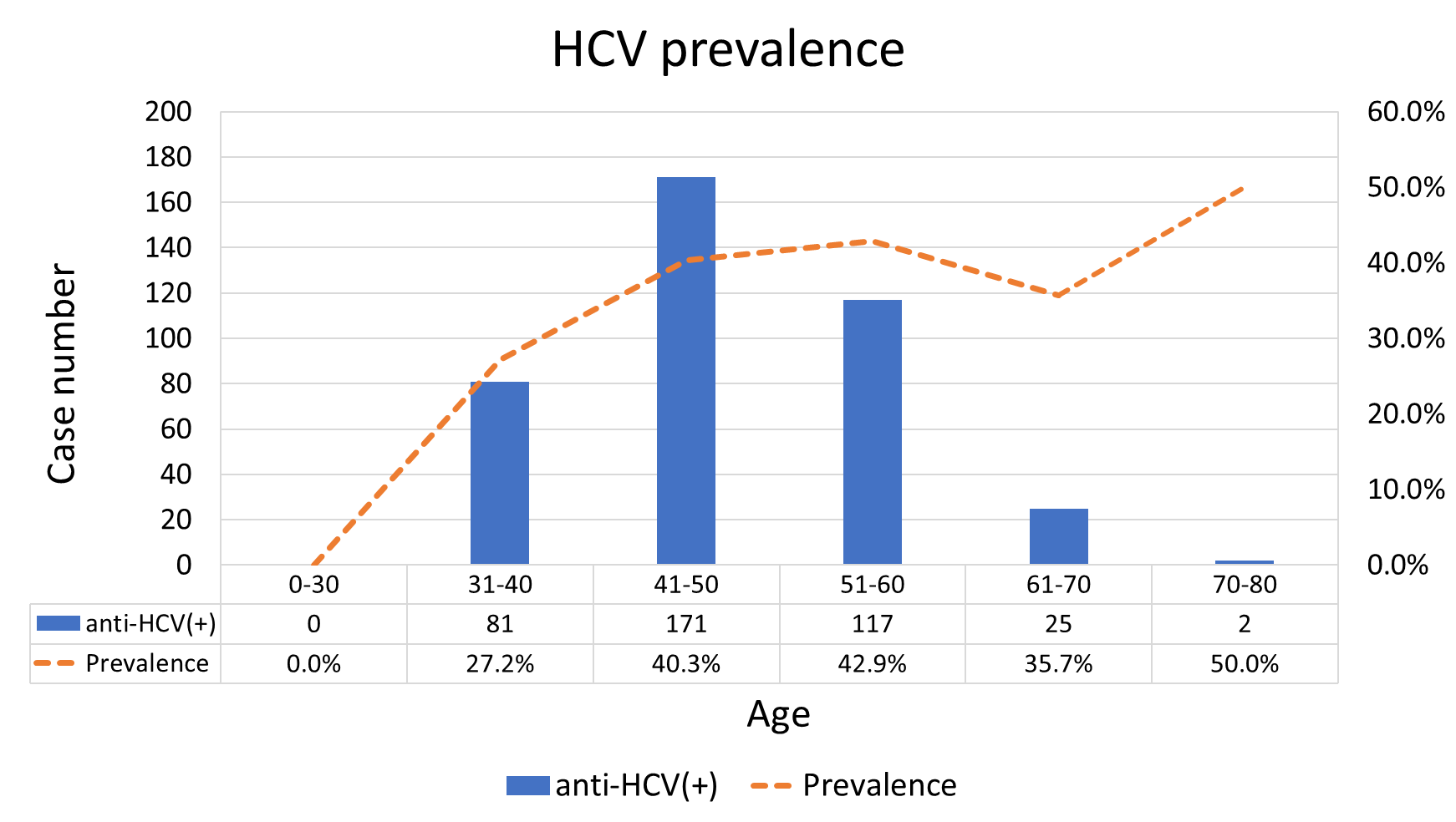


Supplementary figure 3. The distribution of HCV genotypes


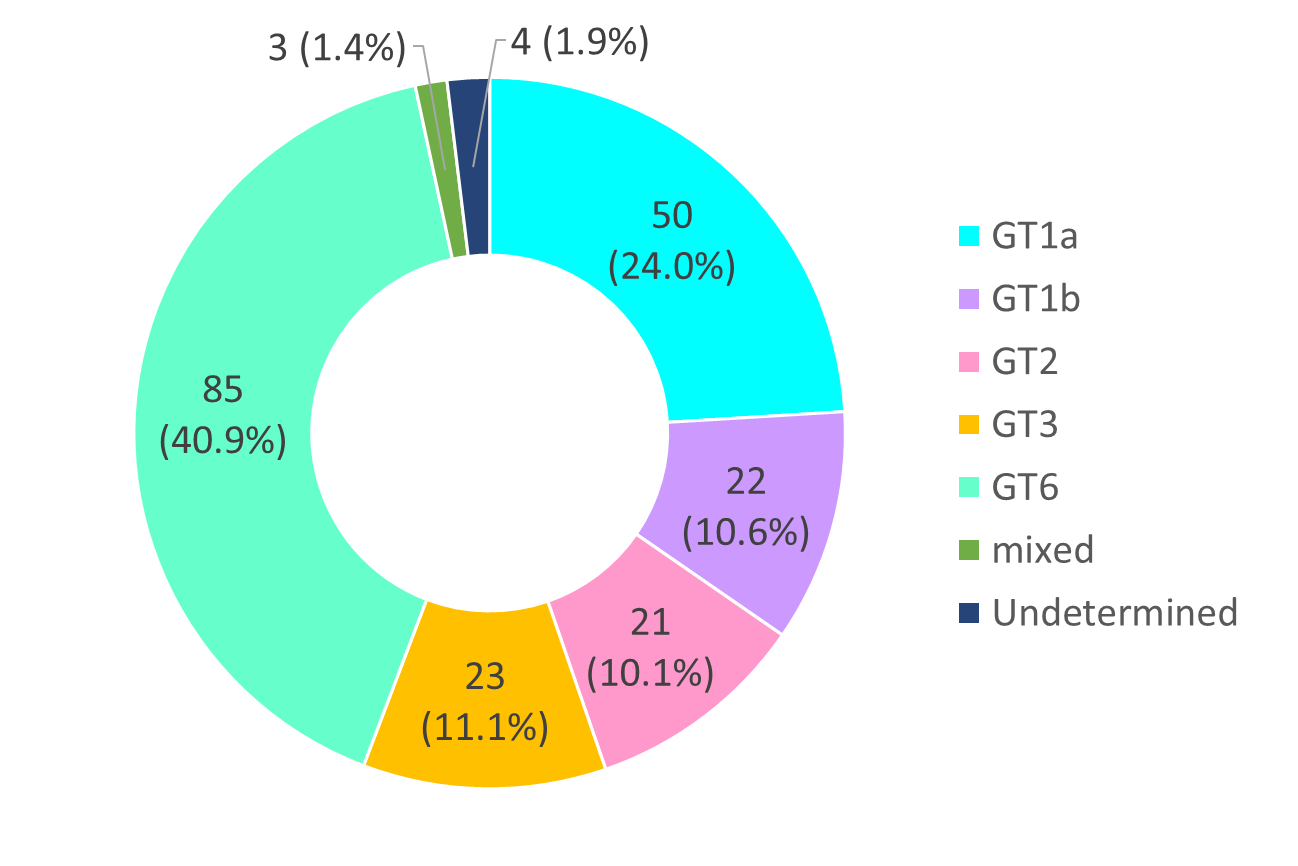


Supplementary Figure 4. The changing prevalence of hepatitis among injecting drugs users in Taiwan


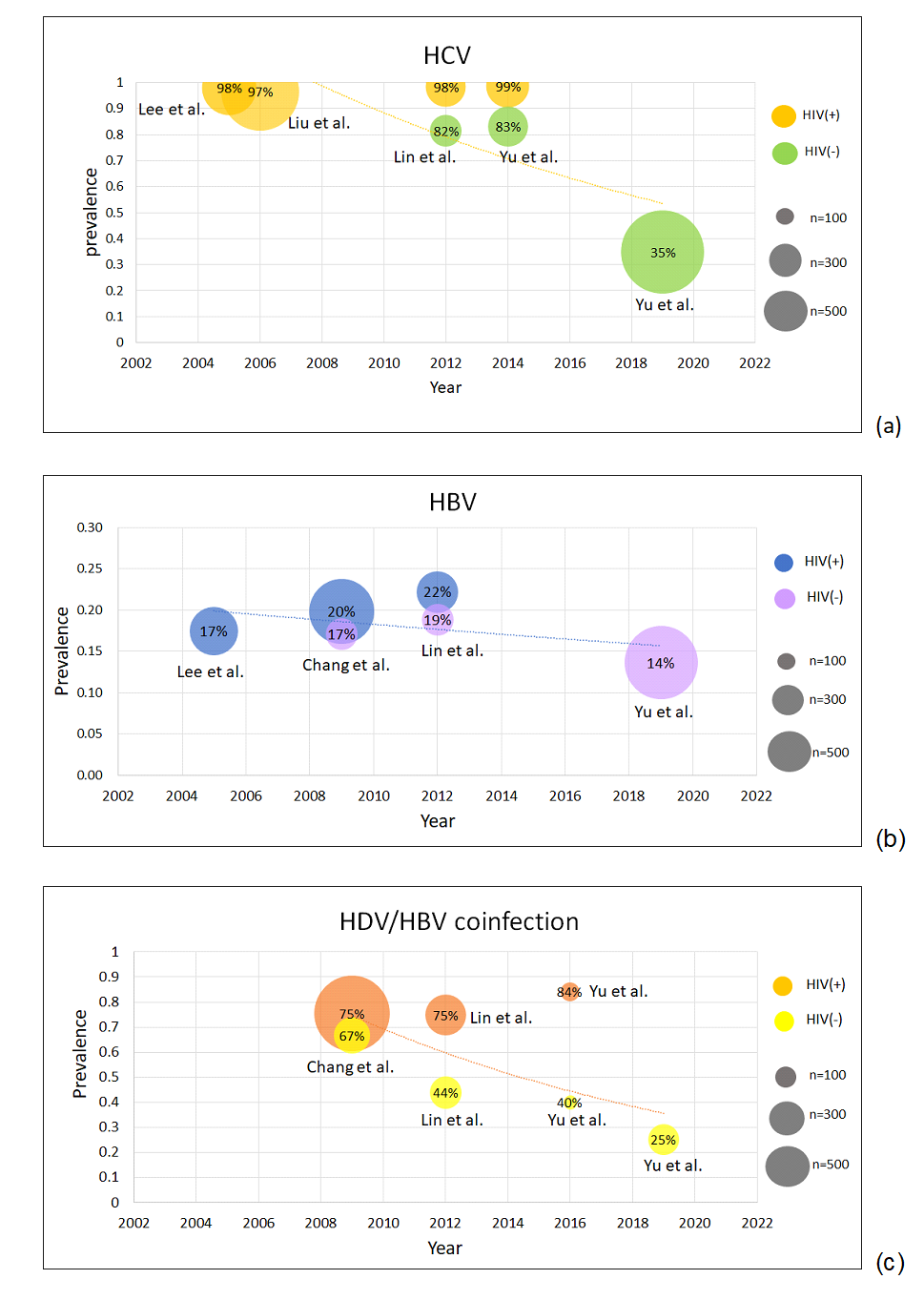


Note: The bubble plot displayed the changing prevalence of hepatitis B, C and D among injecting drug users in Taiwan from 2005 to 2020. The size of bubble represents the case number in each study. The detail information of each study was shown in the supplementary table 1.

Supplementary table 1. The prevalence of hepatitis among injecting drugs users in Taiwan

| **Hepatitis** | **Year** | **n** | **Prevalence** | **HIV** | **Author** | **Reference** |
| --- | --- | --- | --- | --- | --- | --- |
| **HCV** | 2005 | 484 | 97.9% | (+) | Lee et al. | J Formos Med Assoc, 2008 May;107(5):404-11 |
|  | 2006 | 990 | 96.6% | (+) | Liu et al. | Clin Infect Dis, 2008 Jun, 46: 1761–1768 |
|  | 2012 | 263 | 98.3% | (+) | Lin et al. | Hepatology, 2015 Jun;61(6):1870-9. |
|  | 2012 | 164 | 81.5% | (-) | Lin et al. | Hepatology, 2015 Jun;61(6):1870-9. |
|  | 2014 | 297 | 98.7% | (+) | Yu et al. | PLoS One, 2014 Apr 10;9(4): e94791 |
|  | 2014 | 265 | 83.0% | (-) | Yu et al. | PLoS One, 2014 Apr 10;9(4): e94791 |
|  | 2019 | 1137 | 34.8% | (-) | Yu et al. | This study |
| **HBV** | 2005 | 497 | 17.4% | (+) | Lee et al. | J Formos Med Assoc, 2008 May;107(5):404-11 |
|  | 2009 | 904 | 19.8% | (+) | Chang et al. | [J Clin Microbiol. 2011 Mar; 49(3): 1083–1089](https://www-ncbi-nlm-nih-gov.ezp.kmu.edu.tw/pmc/articles/PMC3067682/) |
|  | 2009 | 211 | 17.1% | (-) | Chang et. al | [J Clin Microbiol. 2011 Mar; 49(3): 1083–1089](https://www-ncbi-nlm-nih-gov.ezp.kmu.edu.tw/pmc/articles/PMC3067682/) |
|  | 2012 | 369 | 22.2% | (+) | Lin et al. | Hepatology, 2015 Jun;61(6):1870-9 |
|  | 2012 | 218 | 18.8% | (-) | Lin et al. | Hepatology, 2015 Jun;61(6):1870-9 |
|  | 2019 | 1137 | 13.6% | (-) | Yu et al. | This study |
| **HDV/HBV** | 2009 | 904 | 75.4% | (+) | Chang et al. | [J Clin Microbiol. 2011 Mar; 49(3): 1083–1089](https://www-ncbi-nlm-nih-gov.ezp.kmu.edu.tw/pmc/articles/PMC3067682/) |
| **coinfection** | 2009 | 211 | 66.7% | (-) | Chang et al. | J Clin Microbiol. 2011 Mar; 49(3): 1083–1089 |
|  | 2012 | 263 | 74.9% | (+) | Lin et al. | Hepatology, 2015 Jun;61(6):1870-9 |
|  | 2012 | 164 | 43.9% | (-) | Lin et al. | Hepatology, 2015 Jun;61(6):1870-9 |
|  | 2016 | 57 | 84.2% | (+) | Yu et al. | Kaohsiung J Med Sci. 2016 Oct;32(10):526-530 |
|  | 2016 | 30 | 40.0% | (-) | Yu et al. | Kaohsiung J Med Sci. 2016 Oct;32(10):526-530 |
|  | 2019 | 155 | 25.2% | (-) | Yu et al. | This study |
